# New Appliances and Things Medical

**Published:** 1896-06-06

**Authors:** 


### NEW APPLIANCES AND THINGS MEDICAL.

[We shall be glad to receive, at our Office, 428, Strand, London, W.C., from the manufacturers, specimens of all new preparations and appliances, which may be brought out from time to time.]

### LIQUOR CARNIS (CAFFYN'S).

(Liquor Carnis Company, Aston-Clinton, Bucks.)

We have before had occasion to draw attention to this very excellent meat juice. Its merits are already known to the public, as well as to the profession, and its value as a nutritive and nitrogenous invalid food has been proved over and over again by the most useful of all tests, namely, that of clinical experience. Liquor Carnis is stated to be simply the expressed juice of selected meat, and as such is clearly a highly-digestible proteid food, containing all the nutritive properties of the meat itself, without any of the fibrous and fatty elements which render the mother substance obnoxious to the delicate or physiologically inactive stomach. In anæmia, chlorosis, gastric ulcer, and allied conditions, the patient experiences the greatest benefit when the daily requirements of proteid food stuffs take the form of expressed meat in preference to any other form, the casein of milk not excepted. Meat does not coagulate in heavy masses in the stomach as is the case with milk, but is readily converted into albumose and peptone, and as such absorbed directly from the stomach without further calls upon the digestive powers of the patient.

### COFFEE ESSENCE.

(Messrs. Bromley, Bloomsbury Works, Leeds.)

Bromley's "Tourist" Coffee Essence is quite the best of its kind that we have tasted. In spite of the extreme convenience in using this form of the favourite beverage essences are not general favourites. This is because the public do not usually recognise in them the true flavour of the berry, nor is the substance or appearance of the liquid at all tempting. To Messrs. Bromley's speciality these objections do not apply, and by means of the "Tourist" Coffee Essence a most agreeable cup of coffee can be obtained in a few minutes. By supplying himself with a bottle of this preparation, the tourist can become independent of the varying, and mostly very indifferent, coffee to be had when *en route*. A palatable cup of tea is easy to obtain, but coffee is another matter, and for this reason Messrs. Bromley's excellent coffee essence will be found most useful. The "Tourist" Coffee Essence is not the only speciality that Messrs. Bromley have to offer to the public. We believe their coffee and chicory preparation finds special favour with those who appreciate the latter flavouring. The very moderate price of all the firm's products adds to the many qualities which recommend them.

